# Insights into the evolutionary origins of clostridial neurotoxins from analysis of the *Clostridium botulinum *strain A neurotoxin gene cluster

**DOI:** 10.1186/1471-2148-8-316

**Published:** 2008-11-14

**Authors:** Andrew C Doxey, Michael DJ Lynch, Kirsten M Müller, Elizabeth M Meiering, Brendan J McConkey

**Affiliations:** 1 Department of Biology, University of Waterloo, 200 University Avenue West, Waterloo, Ontario, N2L 3G1, Canada; 2 Guelph-Waterloo Centre for Graduate Studies in Chemistry and Biochemistry, University of Waterloo, 200 University Avenue West, Waterloo, Ontario, N2L 3G1, Canada

## Abstract

**Background:**

Clostridial neurotoxins (CNTs) are the most deadly toxins known and causal agents of botulism and tetanus neuroparalytic diseases. Despite considerable progress in understanding CNT structure and function, the evolutionary origins of CNTs remain a mystery as they are unique to *Clostridium *and possess a sequence and structural architecture distinct from other protein families. Uncovering the origins of CNTs would be a significant contribution to our understanding of how pathogens evolve and generate novel toxin families.

**Results:**

The *C. botulinum *strain A genome was examined for potential homologues of CNTs. A key link was identified between the neurotoxin and the flagellin gene (CBO0798) located immediately upstream of the BoNT/A neurotoxin gene cluster. This flagellin sequence displayed the strongest sequence similarity to the neurotoxin and NTNH homologue out of all proteins encoded within *C. botulinum *strain A. The CBO0798 gene contains a unique hypervariable region, which in closely related flagellins encodes a collagenase-like domain. Remarkably, these collagenase-containing flagellins were found to possess the characteristic HEXXH zinc-protease motif responsible for the neurotoxin's endopeptidase activity. Additional links to collagenase-related sequences and functions were detected by further analysis of CNTs and surrounding genes, including sequence similarities to collagen-adhesion domains and collagenases. Furthermore, the neurotoxin's HCRn domain was found to exhibit both structural and sequence similarity to eukaryotic collagen jelly-roll domains.

**Conclusion:**

Multiple lines of evidence suggest that the neurotoxin and adjacent genes evolved from an ancestral collagenase-like gene cluster, linking CNTs to another major family of clostridial proteolytic toxins. Duplication, reshuffling and assembly of neighboring genes within the BoNT/A neurotoxin gene cluster may have lead to the neurotoxin's unique architecture. This work provides new insights into the evolution of *C. botulinum *neurotoxins and the evolutionary mechanisms underlying the origins of virulent genes.

## Background

Clostridial neurotoxins (CNTs) are the most poisonous biological toxins known and molecular agents of botulism and tetanus neuroparalytic diseases [1]. Due to their extreme toxicity and potential threat as bioterrorism agents, they are listed as Category A agents by the Centers for Disease Control and Prevention along with other deadly agents such as anthrax. Elucidating the mechanisms by which CNTs evolved is therefore of significant importance to our understanding of pathogen evolution and emerging diseases.

While considerable progress has been made in understanding CNT structure and function [2-9], like many toxins and virulence factors, the evolutionary origins of CNTs are unclear. CNTs are produced by four phylogenetically distinct groups (I-IV) of *C. botulinum*, and also by strains of *C. tetani*, *C. baratii*, and *C. butyricum *[10]. As demonstrated by the scattered phyletic distribution of neurotoxin-producing clostridia [10] and the patterns of sequence similarity between different neurotoxin gene clusters [11], CNT genes appear to have undergone significant lateral transfer between different species of *Clostridium*. The occurrence of lateral transfer is also supported by the discovery of plasmid-encoded neurotoxin genes in numerous *C. botulinum *strains [12], as well as the existence of putative insertion sequences flanking the neurotoxin gene cluster [13].

While CNTs have undergone frequent lateral transfer between species of *Clostridium*, no CNT homologues have been identified outside of the *Clostridium *genus. CNTs form an isolated protein family according to SCOP [14] and PFAM [15] and have a unique structural architecture that complicates the identification of related proteins and potential ancestors. While CNT domains have little detectable sequence similarity to proteins outside of the CNT family, there are however some structural and functional similarities to other domain families. The beta-trefoil, a three-fold symmetrical structure that forms the C-terminal receptor binding domain (HCRc) and associated hemagglutinin components, is common to interleukins, ricin-like lectins, and fibroblast growth factors [16]. The adjacent HCRn domain, also involved in receptor binding, forms a jelly-roll like structure similar to laminin globular G domains [4]. The central translocase adopts a long alpha-helical structure containing alpha-helical bundles that resemble those found in translocase-like domains of other toxins [17]. Lastly, the N-terminal catalytic domain has been grouped under the zincin-like group of metalloproteases by SCOP and under the Peptidase MA clan by the MEROPS database [18]. It contains a HEXXH zinc-binding motif found in other zinc endopeptidases, but has only weak structural similarity to other members of the Peptidase MA clan [5].

Diversity of domain and fold composition and extreme sequence divergence are common features of bacterial toxins [19]. Rapid sequence evolution in toxin genes is largely a consequence of the evolutionary 'arms race' between pathogen and host [19]. Therefore, it is important to consider that evolutionarily related toxins may only share weak sequence similarity and may have undergone considerable structural rearrangements. Here, we present evidence supporting the hypothesis that CNTs were formed within an ancestral *Clostridium *species by duplications and rearrangements of neighboring genes within the neurotoxin gene cluster, and identify the likely evolutionary precursors of CNTs and surrounding genes. Multiple links to collagenase-related sequences and functions are detected through an analysis of the nearby flagellin and hemagglutinin genes (strain A) in addition to the CNT domains. The detected links provide novel insights into the evolutionary origins and ancestral function of the neurotoxin gene cluster.

## Results and Discussion

### Ancient gene duplications within the BoNT/A neurotoxin gene cluster

**Figure 1:**
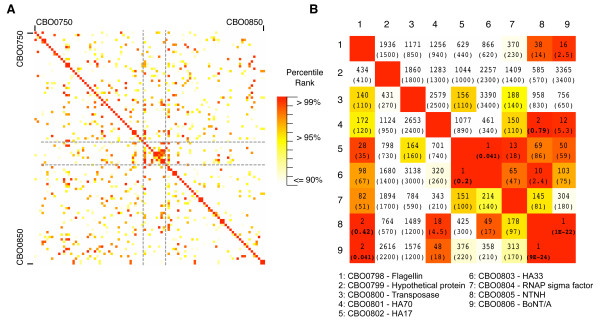
**Protein sequence similarity heat map surrounding the BoNT/A neurotoxin gene cluster**. Sequence similarity scores, *E*-values and percentile ranks were calculated for all pairwise combinations of putative proteins encoded in the *C. botulinum *strain A genome. A) A heat map of the percentile ranks for pairwise alignments involving 100 genes surrounding the neurotoxin gene cluster (described in Methods). B) Similarity ranks and *E*-values (in brackets) for pairwise protein sequence alignments in the neurotoxin gene cluster, corresponding to BoNT/A, NTNH, CBO0798, associated hemagglutinin components and other neighboring genes. *E*-values < 1 are in boldface.

**Figure 2:**
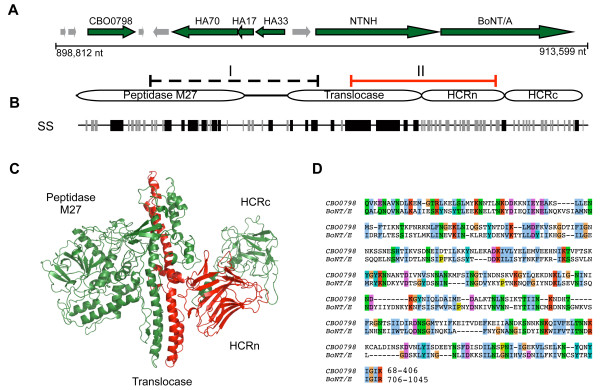
**Genomic location of flagellin CBO0798 and regions of sequence similarity with CNTs**. A) Genomic context of the neurotoxin gene cluster for *C. botulinum *A. str. Hall. B) Domain structure of CNTs and regions of detected similarity with NTNH (region I) and BoNT/A (region 2) according to SSEARCH. A consensus CNT secondary structure based on a multiple sequence alignment is indicated below the schematic, with black lines representing alpha helices and grey lines representing beta sheets. C) The structure of BoNT/A (PDB ID 3BTA) with region II highlighted in red. D) A Smith-Waterman alignment of region II from *C. butyricum *BoNT/E and CBO0798.

A comprehensive analysis of pairwise sequence similarities was performed for all proteins encoded within the *C. botulinum *(strain Hall A, ATCC 3502) genome [20], in an attempt to identify distant homologues of CNTs and possible sequence remnants of the evolutionary process by which CNTs originated. This initial analysis was limited to a single genome for a more sensitive detection of pairwise homologies using a restricted database, however subsequent searches were also performed using all available clostridial genomes. For the 3615 proteins encoded within *C. botulinum *(strain Hall A, ATCC 3502) [20], a 'heat map' of pairwise sequence similarity was constructed (see Methods) (Figure 1). For each pairwise alignment, the *E*-value and percentile rank relative to all other pairwise alignments was calculated using SSEARCH [21]. When compared by percentile rank, the neurotoxin gene cluster stood out as a "hot spot" of local pairwise sequence similarities. The neurotoxin gene cluster can be seen as a distinct cluster of high-scoring pairs in the centre of the heat map region in Figure 1A. Based on both the percentile ranks and *E*-values for the pairwise alignments corresponding to these genes (Figure 1B), there are clear sequence similarities between multiple sequences within this region, including BoNT/A, non-toxic non-hemagglutinin (NTNH), the adjacent hemagglutinin (HA) components and the adjacent CBO0798 gene encoding a flagellin protein (NCBI accession YP_001253335). BoNT/A and NTNH produced the top-scoring alignments with each other out of 3615 proteins in *C. botulinum *strain A (*E *= 1e-22, 9e-24), an expected result given previously identified sequence similarities between BoNTs and NTNH [22] as well as their virtually identical domain architecture as identified by the NCBI's conserved domain database annotation (e.g., for NCBI IDs ABP48106 and BAA90660). Surprisingly, the next highest match in both cases corresponds to the CBO0798 flagellin gene located immediately upstream of the neurotoxin gene cluster (Figure 2A). The associated *E*-values were 0.041 and 0.42 for BoNT/A and NTNH, respectively (Figure 1B). CBO0798 aligned with NTNH and BONT/A in two different CNT regions (I and II) (Figure 2B, Additional Files 1, 2). Additional searches using the sequences of CNTs from other strains also identified CBO0798 as the most consistent top ranked hit out of all *C. botulinum *strain A proteins with sequence identities between CNTs and CBO0798 ranging from 20–24%, and the strongest alignments involving region II of CNTs (Additional File 1).

In addition to the detected similarities between CBO0798-BoNT/A and NTNH-BoNT/A, sequence similarities were also detected between the beta-trefoil hemagglutinin components (HA33 and HA17). HA33 and HA17 were identified as reciprocal top ranked matches (*E *= 0.041, 0.2), and a weak similarity was detected between HA33 and the C-terminal (beta-trefoil) regions of NTNH (ranked 10th, *E *= 2.4). Sequence similarity was also found between the hemagglutinin components HA70 and residues 39–474 of NTNH (ranked #2 out of all pairwise alignments with HA70 as the query, *E *= 0.72). Though the *E*-values calculated above are not all statistically significant, the high-ranking scores relative to the 3615 *C. botulinum *proteins suggest that multiple genes within the BoNT/A neurotoxin gene cluster are likely distant homologues that have undergone extensive sequence divergence.

### Sequence similarity to the upstream flagellin gene

To identify other clostridial sequences homologous to CBO0798, a PSI-BLAST [23] search was conducted starting with the CBO0798 sequence (default parameters, results restricted to *Clostridia*). All homologues identified in the first iteration were members of the flagellin family. The second iteration identified additional flagellins, followed by the type E botulinum toxin (BoNT/E) from *C. butyricum *with an *E*-value of 0.05 [23% sequence identity over residues 88–406 of flagellin and 727–1045 (region II) of BoNT/E, see Additional File 2 for the alignment]. To check for the influence of composition on the alignment, two permutation reshuffling tests were performed, which calculate the probability that random sequences of the same composition could result in similar alignment scores. The permutation reshuffling tests detected significant sequence similarity between the two proteins with (p = 0.0024) and without (p = 0.011) statistically overrepresented amino acids included (see Methods).

According to the sequence alignments produced by PSI-BLAST and SSEARCH, the region of CNTs with the strongest detected similarity to CBO0798 (region II) includes most of the translocase domain as well as the HCRn domain (Figure 2B–D). Region I was also detected by SSEARCH (Additional File 1), spanning the peptidase and 'belt' region, though without definitive statistical significance (*E *= 0.41). The translocase, an extended alpha-helical domain, has a general structural similarity to the central helical regions of known flagellin structures (see PDB IDs 1io1, 2zbi, 2d4x). The beta-rich domains of flagellin are highly variable however, and it is this variable region of flagellin that shares similarity with the HCRn domain of CNTs. As a structure is not available for the variable region of CBO0798, 3D-PSSM [24] was used to predict the fold of CBO0798's central region. The structure for the CNT's HCRn jelly-roll domain was the top ranked structural match for this region (*E *= 0.34), additionally supporting homology between the two proteins.

CBO0798 is annotated in the NCBI database as a member of the flagellar hook associated protein 3 (FlgL) family. This flagellin gene has been mentioned in previous CNT studies due to its close proximity to the neurotoxin gene cluster [13] and its existence in numerous *C. botulinum *type A strains and associated plasmids [25]. Flagellins are also known to have key roles in the virulence of bacterial pathogens [26], have been shown by mass spectrometry studies to interact with CNT components [27], and possess previously unreported common structural features with CNTs (i.e., both contain a central region composed of extended alpha-helices followed by beta-rich domains [4,28]). These additional functional and structural links further support a potential evolutionary relationship between CBO0798 and CNTs.

### Collagenase-like domains in the flagellin hypervariable region

Comparative sequence analysis of CBO0798 was performed by aligning CBO0798 to other flagellins from *Clostridium *species (Additional File 3). According to the alignment, CBO0798 has a highly divergent central region containing a unique insert (residues ~135–360), and this insert region comprises a large portion of CBO0798's alignments with CNTs. The existence of a unique central region within CBO0798 is not surprising, since flagellins are known to contain conserved regions at the N- and C-terminus but have a hypervariable central region that is structurally exposed on the flagellar surface [26]. As the structurally exposed region of the flagellar filament, the hypervariable region can interact with the host cell and is thus critical to flagellin-mediated virulence [26]. Interestingly, it is the variable region of CBO0798 that is central to the CBO0798-CNT alignments and that was predicted by 3D-PSSM to possess a jelly-roll fold similar to HCRn.

**Figure 3:**
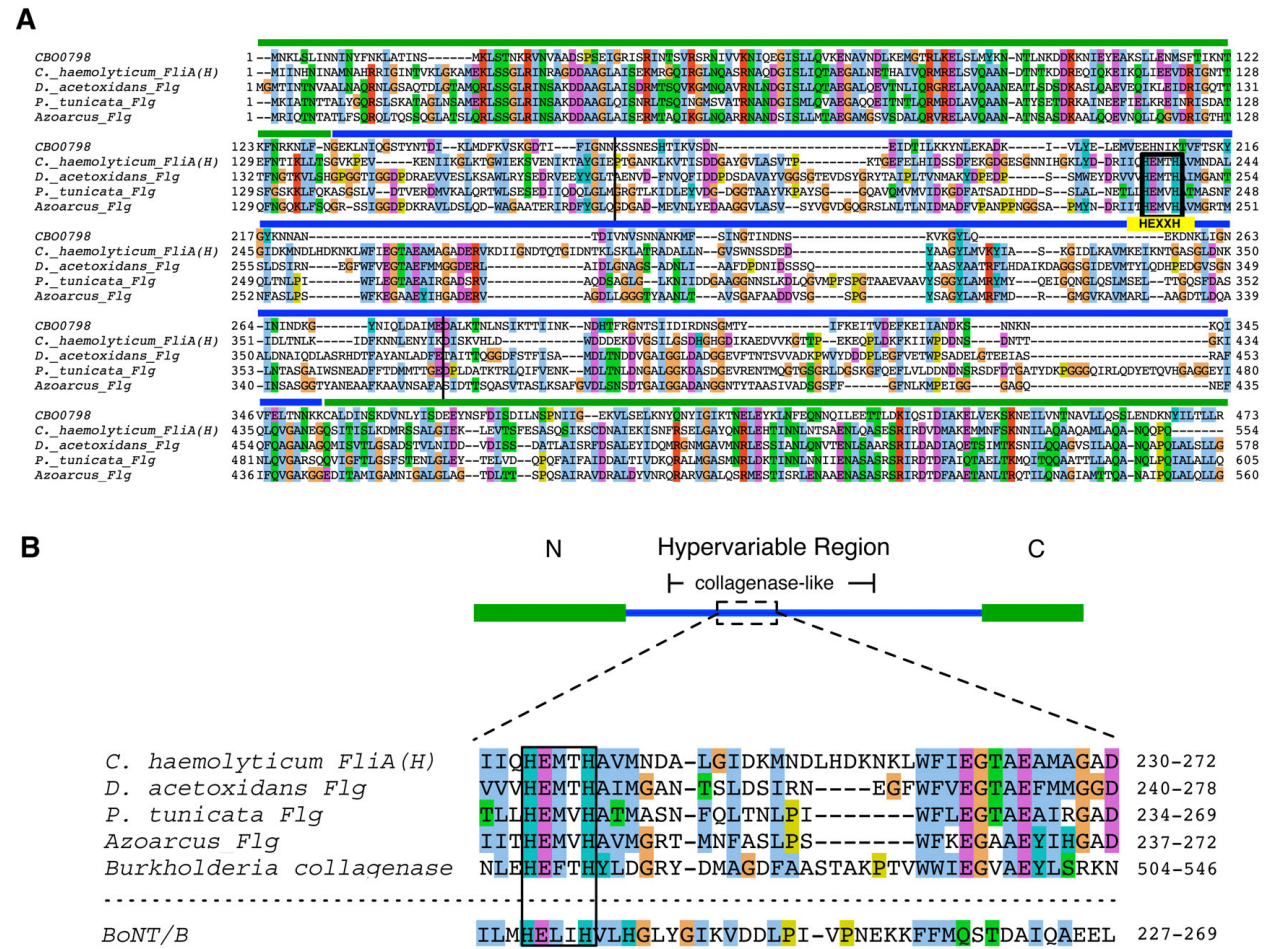
**Collagenase-like sequences within the flagellin hypervariable region**. A) A multiple alignment of CBO0798 and collagenase-containing flagellins identified by PSI-BLAST. Vertical black bars in the alignment correspond to the collagenase-containing region identified by a PSI-BLAST search using *C. haemolyticum *FliA(H) as the query. B) A schematic of a representative collagenase-containing flagellin based on the FliA(H) sequence. An alignment of similar HEXXH-containing segments from BoNT/B, a microbial collagenase, and the collagenase-containing flagellins are shown below the schematic. Accession numbers are provided in the Methods.

To characterize the origins of the insert, we examined similarly located inserts identified within the hypervariable region of a small number of additional flagellins from *Clostridium *species (Additional File 3). While the sequences within the hypervariable region are highly divergent from one another as expected, one insert in particular [the insert of FliA(H) from *C. haemolyticum*] was identified to be both the largest insert and the only insert region with detected homology to other proteins using PSI-BLAST. FliA(H) is a relatively close homolog of CBO0798, as FliA(H) was the only flagellin detected using CBO0798's C-terminal region (residues 114–452) as a BLAST query sequence (*E *= 0.076). A PSI-BLAST search revealed that the hypervariable region of FliA(H) possesses significant similarity to microbial collagenases (*E *= 8e-04, iteration 2) and to the hypervariable regions of several flagellins from non-clostridial species (Figure 3). Remarkably, both the detected microbial collagenases and collagenase-like regions within the identified flagellins contain a HEXXH motif, the critical catalytic residues responsible for the CNT's zinc-endopeptidase activity. The alignment of CBO0798 with collagenase-containing flagellins and alignment of the HEXXH-containing segments from these flagellins, BoNT/B, and a representative microbial collagenase are shown in Figure 3.

The identified link to collagenase sequences by analysis of the flagellin hypervariable region is a striking result given the strong similarities between collagenases and the CNT's Peptidase M27 domain. Both collagenases (Peptidase M9s) and Peptidase M27's are zinc-endopeptidases and are grouped under the same peptidase family (thermolysin-like Peptidase MA clan) by the MEROPS database [18]. As exotoxins, collagenases play a major role in clostridial toxicity by degrading collagenous host tissues [29,30]. For instance, *C. perfringens*, a species responsible for clostridial myonecrosis (gas gangrene), produces a tissue-degrading collagenase known as kappa-toxin [30]. Collagenases are therefore an excellent candidate evolutionary precursor of CNTs as both collagenases and CNTs function as clostridial toxins, and both share the same fundamental proteolytic mechanism.

As the hypervariable region encodes the outer exposed portion of the flagellin filament, it would be ideally situated to interact with (and potentially degrade) host cell wall components such as collagen. The identified sequences may therefore encode a novel family of virulent flagellins with collagenase activity. Future experimental verification of this predicted activity would be valuable, and could potentially lead to new avenues of research concerning the virulent functions of bacterial flagellins.

### Additional evidence of collagenase-related functions within the neurotoxin gene cluster

Several additional links to collagenases and collagen-related domains were detected for other sequences present within the BoNT/A neurotoxin gene cluster. All sequenced *Clostridium *genomes were screened for potential homologues of each of the BoNT/A neurotoxin gene cluster components. In a dataset of over 55000 sequences, a search of BoNT/A detected flagellin as the third top ranked hit outside of the CNT family (*E *= 0.23). While HA33 expectedly displayed similarities with other ricin-like components (e.g., a ricin-domain from a *C. acetobutylicum *cellulase, NP_347343, *E *= 0.019), HA70 displayed the strongest similarity to *C. perfringens *enterotoxin (YP_697710, *E *= 0.0042) followed by *C. tetani *collagenase (NP_783761, *E *= 0.22). A HEXXH binding motif was also identified within this collagenase sequence. A PSI-BLAST search of flagellin CBO0798 restricted to the *Clostridium *genus also detected collagen-adhesion proteins with alignments spanning the hypervariable region after three iterations (*E *= 0.017, ZP_02635881). This result is consistent with the analysis linking CBO0798 with flagellins containing collagenase-like hypervariable regions.

**Figure 4:**
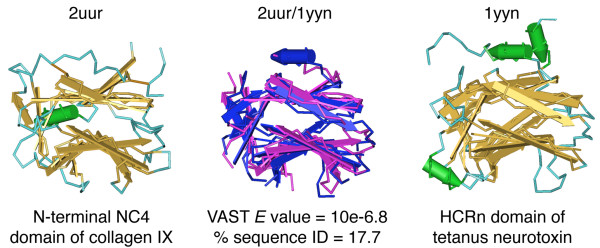
**Structural similarity between HCRn and the NC4 domain of collagen IX**. A structural superposition of the human collagen IX NC4 domain (2UUR) and the TeNT HCRn domain (1YYN) was performed using the VAST alignment algorithm http://www.ncbi.nlm.nih.gov/Structure/VAST/vastsearch.html. In the structural alignment 2UUR and 1YYN are colored pink and blue respectively.

Another key result was obtained when examining sequence and structural similarities between the HCRn domain and the full NCBI nr database, including eukaryotic sequences. After two iterations starting with BoNT/A's HCRn domain, PSI-BLAST detected a region of chicken type XII collagen (AAA48635, *E *= 0.03). The detected sequence similarity occurred with collagen's thrombospondin N-terminal like domains. Recently, the structure of this family of domains has been determined for the NC4 domain of collagen IX [31]. The fold of NC4 (PDB ID 2UUR) is remarkably similar to that of HCRn (Figure 4). To determine the extent of structural similarity between these two domains, we analyzed structural neighbours of the NC4 domain using the VAST structural alignment algorithm [32]. Sorted by VAST *E*-value, the two most structurally similar domains to PDB ID 2UUR were its identified fold family (PDB IDs 2ES3 and 2OUJ), the thrombospondin N-terminal domain), followed by the HCRn domain (PDB ID 1DLL) of the tetanus neurotoxin (*E *= 10e-9.9). Ranked by sequence similarity based on structural alignments, the tetanus HCRn domain (PDB ID 1YYN) ranked first out of all known structures in the Protein Data Bank (%ID = 17.7).

As the detected similarities between the HCRn domain and the collagen NC4 domain occur across kingdoms, this may represent an instance of structural mimicry rather than a direct evolutionary relationship. Given the multiple identified links to collagenases, and that structural mimicry of collagen has been proposed as a mechanism for other collagenase enzymes [33], the link between HCRn and the collagen NC4 domain may be indicative of a similar mechanism. A role in collagen-binding is entirely possible for CNTs as previous studies have shown that expression of TeNT enhances adhesion of epithelial cells to collagen, laminin, and fibronectin [34]. While the observed similarities support the hypothesis of convergent evolution and structural mimicry, the possibility that HCRn was transferred to *Clostridium *from a eukaryotic source cannot be completely ruled out. This scenario has been demonstrated recently for the *Clostridium *glyceraldehyde-3-phosphate dehydrogenase gene [35].

## Conclusion

A comprehensive search was conducted for potential distant homologues of CNTs, starting with a genomic analysis of *C. botulinum *strain A, followed by a more general search involving additional clostridial and eukaryotic species. Multiple independent links to collagenase-related sequences were identified, including the detected similarities involving the upstream flagellin gene (CBO0798) in the BoNT/A neurotoxin gene cluster, distant BLAST hits to collagenase-related domains, and detected structural similarities to the collagen NC4 domain. As microbial collagenases are phyletically widespread compared to CNTs, they represent a protein family likely to be ancestral to CNTs. Given this and the multiple detected links to collagenase-related sequences, it is proposed that the ancestral function of the neurotoxin gene cluster may have been related to collagen binding and degradation, a hypothesis that places CNT sequence, structure, and function within the broader context of other clostridial toxins and the evolution of clostridial pathogenesis.

The CBO0798 flagellin gene appears to be a divergent member of a unique class of flagellins containing a collagenase-like hypervariable domain, an ideal arrangement for the development of novel virulent functions and co-evolution with host cell walls. It is possible that repeats and rearrangements of a gene ancestral to CBO0798 may have been involved in the origin of the ancestral CNT gene. To date, the CBO0798 flagellin sequence has only been identified in a number of group I strains (see [25]), and identification and analysis of additional CBO0798 homologues in other strains could provide a broader context to the evolutionary relationship between flagellins, collagenases, and CNTs.

## Methods

### Sequence dataset and database searches

Botulinum neurotoxins A-G (P10845, ABM73983, BAA08418, AAB24244, CAA43999, 1904210A, CAA52275), and NTNH/A (YP_001253341) sequences were retrieved from NCBI. The flagellin and collagenase sequences used in the alignment of the HEXXH-containing segment (Figure 3) were *Clostridium haemolyticum *flagellin [FliA(H)], BAB87738; *Pseudoalteromonas tunicata *flagellin, ZP_01132756; *Azoarcus *BH72 flagellin, YP_934037; *Desulfuromonas acetoxidans *flagellin, ZP_01312630; and *Burkholderia pseudomallei *collagenase, ZP_01765667. The following default parameters were used in all PSI-BLAST [23] searches unless specified otherwise: Blosum62 matrix, Gap existence: 11, Gap Extension: 1, *E*-value cutoff = 0.005, with conditional compositional matrix score adjustment. The SSEARCH [21] program from the FASTA package (version 3.515) was used to search the *C. botulinum *protein database, and was obtained via the SANGER website ftp://ftp.sanger.ac.uk/pub/pathogens/cb/. SSEARCH was run with default parameters, except for the -z 11 flag, which computes the regression by reshuffling the target sequence library (removing the influence of homologous sequences present within the genome). For searching additional *Clostridium *species, the following protein sequence databases were retrieved from the NCBI FTP server: *C. acetobutylicum *ATCC 824, *C. beijerinckii *NCIMB 8052, *C. botulinum *A ATCC 3502, *C. botulinum *A ATCC 19397, *C. botulinum *A Hall, *C. botulinum *A3 Loch Maree, *C. botulinum *B1 Okra, *C. botulinum *F Langeland, *C. difficile *630, *C. kluyveri *DSM 555, *C. noyvi *NT, *C. perfringens *13, *C. perfringens *ATCC 131245, *C. perfringens *SM101, *C. phytofermentans *ISDg, *C. tetani *E88, *C. thermocellum *ATCC 27405.

### Construction of sequence similarity heat map

A perl program was written to generate a 2D sequence similarity matrix based on all-against-all Smith-Waterman alignment scores using 3615 sequences from the *C. botulinum *protein database. Proteins were ranked by *E*-values computed by the SSEARCH program with default parameters. The matrix consists of query sequences on the Y-axis, target database proteins on the X-axis, and data values correspond to percentile ranks. This approach was used to detect distant pairwise similarities within gene clusters that may reflect ancient gene duplication blocks. The matrix was visualized using Treeview version 1.1.1 http://rana.lbl.gov/EisenSoftware.htm.

### Permutation testing

The PRSS component of the FASTA package [21] was used for sequence reshuffling and the permutation test. The permutation reshuffling test calculates the optimal Smith-Waterman alignments of the first query sequence with N reshuffled versions of the second query sequence. The alignment score of the unshuffled sequences is compared to the distribution of scores obtained using the reshuffled query sequence, which is fit to an extreme value distribution. From this distribution, the probability that the observed alignment score could have resulted from a random sequence of the same composition is estimated. Default parameters were used and 1000 reshuffled sequences were used to generate the random distribution of alignment scores.

To detect potential compositional bias, the composition of CNTs and CBO0798 was analyzed relative to all protein sequences in *C. botulinum *strain A as a reference. One amino acid type, asparagine, was found to be significantly elevated in both CBO0798 and CNT sequences (Z > 2 standard deviations). To verify that PSI-BLAST hits from CBO0798 to CNTs sequences were not due to composition, all asparagine residues were removed from CBO0798 and the top-scoring alignment detected via PSI-BLAST (*C. butyricum *BoNT/E), and permutation reshuffling tests were repeated using the altered sequences.

## Authors' contributions

A.C.D. designed and performed the analysis and wrote the paper. B.J.M. designed the analysis and co-wrote the paper. E.M.M., M.D.J.L. and K.M.M. assisted with analysis and preparation of the manuscript.

## Supplementary Material

Additional file 1 **Detected sequence similarities between CBO0798 (flagellin) and CNT sequences.** SSEARCH was used to screen the *C. botulinum *A protein database (3615 sequences) plus the target CNTs using default parameters. *E*-values were calculated within SSEARCH using randomly reshuffled copies of the library sequences, as described in Methods. CBO0798's rank relative to all 3615 *C. botulinum *proteins, associated E-value, and the alignment regions are reported for nine search cases (BoNT/A-G, TeNT, and NTNHA). The flagellin aligned to two separate regions in the CNTs, suggesting an ancestral duplication. Click here for file

Additional file 2 **Key alignments between CBO0798 and CNTs produced by SSEARCH and PSI-BLAST. **Smith-Waterman alignments between BoNT/A-CBO0798 and NTNH-CBO0798 were performed using SSEARCH (default parameters used with -z 11 flag). The listed *E*-values are based on a single pairwise alignment of both sequences rather than a database search. The alignment between CBO0798 and BoNT/E from *C. butyricum *is the result of a PSI-BLAST search of CBO0798 (restricted to *Clostridia*) using default parameters with composition-based statistics. Click here for file

Additional file 3 **Multiple alignment of clostridial flagellins.** The N- and C-terminal domains are indicated, and the intermediate section represents the flagellin hypervariable region. The collagenase-like insert identified within the hypervariable region of FliA(H) is boxed in red. CBO0798 is underlined in black. Additional clostridial flagellins containing large hypervariable region inserts are grouped with CBO0798 and FliA(H) at the beginning of the alignment. Click here for file
