# Supplementary material for: Insights into the evolutionary origins of clostridial neurotoxins from analysis of the Clostridium botulinum strain A neurotoxin gene cluster
Source: BMC Evol Biol. 2008 Nov 14;8:316. doi: 10.1186/1471-2148-8-316 (PMC2605760; doi:10.1186/1471-2148-8-316)
Supplement: Additional file 1 — Detected sequence similarities between CBO0798 (flagellin) and CNT sequences. SSEARCH was used to screen the C. botulinum A protein database (3615 sequences) plus the target CNTs using default parameters. E-values were calculated within SSEARCH using randomly reshuffled copies of the library sequences, as described in Methods. CBO0798's rank relative to all 3615 C. botulinum proteins, associated E-value, and the alignment regions are reported for nine search cases (BoNT/A-G, TeNT, and NTNHA). The flagellin aligned to two separate regions in the CNTs, suggesting an ancestral duplication. [file 1471-2148-8-316-S1.pdf]

Additional File 1. Detected sequence similarities between CBO0798 (flagellin) and CNT sequences. SSEARCH was used to screen the *C. botulinum* A protein database (3615 sequences) using default parameters. The [-z 11] flag was used to calculate the regression using randomly reshuffled copies of the library sequences. CBO0798's rank relative to all 3615 *C. botulinum* proteins, associated E-value, and the alignment regions are reported for nine search cases (BoNT/A-G, TeNT, and NTNHA).

Sequence	*E*	Rank	CNT alignment region	Flagellin CBO0798 alignment region	Sequence identity
BoNT/A	0.041	1	676-1113	1-469	20.9%
BoNT/B	3.6	19	715-1110	69-468	22.9%
BoNT/C	0.03	1	676-1079	46-468	22.7%
BoNT/D	0.038	1	675-1075	59-468	23.7%
BoNT/E	0.059	1	690-1045	43-406	23.5%
BoNT/F	5.7	15	674-1118	11-468	23.5%
BoNT/G	5.2	19	727-1147	5-457	22.5%
TeNT	7.8	32	350-845	6-473	19.7%
NTNH	0.42	1	158-562	44-471	24.1%
