# Supplementary material for: Insights into the evolutionary origins of clostridial neurotoxins from analysis of the Clostridium botulinum strain A neurotoxin gene cluster
Source: BMC Evol Biol. 2008 Nov 14;8:316. doi: 10.1186/1471-2148-8-316 (PMC2605760; doi:10.1186/1471-2148-8-316)
Supplement: Additional file 2 — Key alignments between CBO0798 and CNTs produced by SSEARCH and PSI-BLAST. Smith-Waterman alignments between BoNT/A-CBO0798 and NTNH-CBO0798 were performed using SSEARCH (default parameters used with -z 11 flag). The listed E-values are based on a single pairwise alignment of both sequences rather than a database search. The alignment between CBO0798 and BoNT/E from C. butyricum is the result of a PSI-BLAST search of CBO0798 (restricted to Clostridia) using default parameters with composition-based statistics. [file 1471-2148-8-316-S2.pdf]

Additional File 2. Key alignments between CBO0798 and CNTs produced by SSEARCH and PSI-BLAST.

SSEARCH alignment between BoNT/A and flagellin CBO0798 (default parameters used with -z 11 flag). *E*-values are based on a single pairwise alignment of both sequences rather than a database search.

```
s-w opt: 175  Z-score: 83.9  bits: 26.3 E(): 0.0073
Smith-Waterman score: 175; 21.1% identity (59.2% similar) in 488 aa overlap (676-1113:1-469)

         650       660       670       680          690       700
BoNT/A MLYKDDFVGALIFSGAVILLEFIPEIAIPVLGTFALVSYIA--NKVLTVQTIDNALSKRN
                                     .. ..:.. :   ::. :....  . .::
flagel                               MNKLSLINNINYFNKLATINSMKLSTNKRV
                                             10        20        30

          710       720          730            740
BoNT/A EKWDEVYKYIVTNWLAKVNTQI---DLIRKKMKEAL------ENQAEATKAI--------
         .   .   . :  . ....:::..   ... :...:..      :: ..  : .
flagel NVAADSPSEI--GRISRINTSVRSRNIVVKNIQEGISLLQVKENAVNDLKEMGTRLKELS
                40           50        60        70        80

        750       760       770       780       790       800
BoNT/A INYQYNQYTEEEKNNINFNIDDLSSKLNESINKAMININKFLNQCSVSYLMNSMIPYGVK
         . :. :  ....:.::...  .:  ... .:... .: ....:  ... ...:     .:
flagel LMYKNNTLNKDDKKNIEYEAKSLLENMSFTIKNTKFNRKNLFNGEKLN-IQGSTYNTDIK
       90       100       110       120       130        140

        810       820           830           840       850
BoNT/A RLEDFDASLKDALLKYIYDNRGT----LI----GQVDRLKDKVNNTLSTDIPFQLSKYVD
         : :: .:  :..  .: .:...      :     ...: .  : :   .  : .   ..:.
flagel -LMDFKVSKGDTI--FIGNNKSSNESHTIKVSDNEIDTILKKYNLEKADKIVLYELEMVE
        150         160       170       180       190       200

      860          870       880       890       900          910
BoNT/A NQRLLSTFT-EY-IKNIINTSILNLRYESNHLIDLSRYASKINIGSKVN--FDPIDKNQI
         .. . ..:: .:  ::  ::.:.:.  ..:......    . :: .:::.  ..  :.. :
flagel EHNIKTVFTSKYGYKNNANTDIVNVSNNANKMFSIN---GTINDNSKVKGYLQEKDNKLI
          210       220       230       240          250       260

          920       930       940       950       960       970
BoNT/A QLFNLESSKIEVILKNAIVYNSMYENFSTSFWIRIPKYFNSISLNNEYTIINCMENNSGW
          .:....  .. : .::. ...  :...      : :  :. .. .. .::. ...:::
flagel GNININDKGYNIQL-DAIMEDALKTNLNSIKTTIINK--NDHTFRGNTSIID-IRDNSGM
             270        280       290       300       310

               980       990      1000      1010       1020
BoNT/A -----KVSLN-YGEIIWTLQDTQEIKQRVVFKYSQMINISDYIN-RWIFVTITNNRLNN-
            ..... . ::: . ..... :: .::. ..   . .  :: . . . :.... :.
flagel TYIFKEITVDEFKEIIANDKSNNKNKQ-IVFELTNNKKCALDINSKDVNLYISDEEYNSF
       320       330       340       350       360       370

        1030      1040           1050      1060        1070
BoNT/A --SKIYINGRLIDQKPISNLGNIHA-----SNNIMFKLDGCRDTH--RYIWIKYFNLFD-
         : :  .  .: .: .:.: : .     .:.. .::.  ....  .   .  .. .:
flagel DISDILNSPNIIGEKVLSELKNYQNYIGIKTNELEYKLNFEQNNQILEETTLDKIQSIDI
        380       390       400       410       420       430

       1080      1090      1100      1110      1120      1130
BoNT/A -KELNEKEIKDLYDNQSNSGILKDFWGDYLQYDKPYYMLNLYDPNKYVDVNNVGIRGYMY
        ::: ::  ...  : .:. .:..     :. :: : .
flagel AKELVEKSKNEILVN-TNAVLLQS----SLENDKNYILTLLR
        440       450            460       470
```

SSEARCH alignment between NTNH/A and flagellin CBO0798 (default parameters used with -z 11,12,14,15,16 flag). *E*-values are based on a single pairwise alignment of both sequences rather than a database search.

```
s-w opt: 151  Z-score: 64.8  bits: 22.7 E(): 0.081
Smith-Waterman score: 151; 23.6% identity (51.4% similar) in 449 aa overlap (158-562:44-471)

      130       140       150       160       170       180
NTNH   APKSNKKLNSLISSTIPFPYAGYRETNYLSSEDNKSFYASNIVIFGPGANIVENNTVFYK
                                         :. : :  . :::.     :: :. ...
flagel NKLATINSMKLSTNKRVNVAADSPSEIGRISRINTSVRSRNIVV----KNIQEGISLLQV
            20        30        40        50            60

      190       200       210          220        230       240
NTNH   KEDAENGMGTMTEIWFQPFLTYKYDEFYIDPA--IEL-IKCLIKSLYFLYGIKPSDDLVI
       ::.: : .  :      .  : :: .  .   :    ::   : :.... :   :: .  .
flagel KENAVNDLKEMGTRLKELSLMYKNNTLNKDDKKNIEYEAKSLLENMSFT--IK-NTKFNR
     70        80        90       100       110         120

         250       260            270       280              290
NTNH   PYRLRSELENIEYSQLN----IVDLLVSGGIDPKFINTDP-------YWFTDNYFSNAKK
          . .:  ::. :  :    ..:. :: : :  ::...               .:: ...  :
flagel KNLFNGEKLNIQGSTYNTDIKLMDFKVSKG-DTIFIGNNKSSNESHTIKVSDNEIDTILK
        130       140       150        160       170       180

               300       310       320        330       340
NTNH   VFE----DHRNIYETEIEGNNAIGNDIKLRLKQKFRININ-DIWELNLNYFSKEFSIMMP
        ..    :.  .:: :.  .. : .   .  :   .. : : :: ... :  .: :::
flagel KYNLEKADKIVLYELEMVEEHNIKT--VFTSKYGYKNNANTDIVNVS-NNANKMFSINGT
         190       200       210         220       230        240
```

```
      350       360       370       380             390       400
NTNH   DRFNNALKHFYRKQYYKIDYPENYSINGFVNGQINA------QLSLSDRNQDIINKPEE-
          :. .: . ...  :.    : . .:. : :..:      . .:.. .  :::: ..
flagel INDNSKVKGYLQEKDNKLIGNININDKGY-NIQLDAIMEDALKTNLNSIKTTIINKNDHT
            250       260       270        280       290       300

                   410       420       430       440       450
NTNH   ------IINLLNGNNVSLMRSNIYGDGLKSTVDDFYSNYKIPYNRAYEYHFNNSNDSSLD
             ::.. ...... . ..:  : .:  . .  :: :   :.   ....:..  .::
flagel FRGNTSIIDIRDNSGMTYIFKEITVDEFKEIIANDKSNNK---NKQIVFELTNNKKCALD
             310       320       330       340          350

             460          470         480        490       500
NTNH   ----NVNIGVID---NIPEIIDV--NPYKENCDKFSPVQKITSTREINTN-IPWPINYLQ
           .::. . :   :  .: :.  .:   .   .: ...  .   :.:: . . .:. :
flagel INSKDVNLYISDEEYNSFDISDILNSPNIIGEKVLSELKNYQNYIGIKTNELEYKLNFEQ
      360       370       380       390       400       410

         510       520         530       540       550       560
NTNH   AQNTNNEKFSLSSDFVEVVSSKD--KSLVYSFLSNVMFYLDSIKDNSPIDTDKKYYLWLR
           ::. .  ..  .. ..: :  : :: .  ....   ...  .: ...::.: : :
flagel ----NNQILEETT--LDKIQSIDIAKELVEKSKNEILVNTNAVLLQSSLENDKNYILTLL
          420         430       440       450       460       470
```

PSI-BLAST detection of *C. butyricum* BoNT/E from flagellin CBO0798
(composition-based statistics, *Clostridia-restricted database*)

```
>sp|P30995|BXE_CLOBU  Botulinum neurotoxin type E precursor (BoNT/E) (Bontoxilysin-E)
[Contains: Botulinum neurotoxin E light chain; Botulinum
neurotoxin E heavy chain]
 emb|CAA43998.1|  botulinum neurotoxin [Clostridium butyricum]
Length=1251

 Score = 37.9 bits (87),  Expect = 0.050, Method: Composition-based stats.
 Identities = 82/345 (23%), Positives = 136/345 (39%), Gaps = 52/345 (15%)

Query  88    SLMYKNNTLNKDDKKNIEYEAKSLL----ENMSFTIKNTKFNR-KNLFNGEKLN-IQGST  141
             +L  KN   NK D + IE E    +     N+   +  +  +    L N  K+N ++
Sbjct  727   TLEEKNELTNKYDIEQIENELNQKVSIAMNNIDRFLTESSISYLMKLINEVKINKLREYD  786

Query  142   YNTDIKLMDFKVSKGDTIFIGNNKSSNESHTIKVSDNEI---------DTILKKYNLEKA  192
              N    L+D+ +  G    +G ++    S  I   +N I         D IL  Y  +
Sbjct  787   ENVKTYLLDYIIKHGS--ILGESQQELNSMVIDTLNNSIPFKLSSYTDDKILISYFNKFF  844

Query  193   DKIVLYELEMVEEHNIKTVFTSKYGYKNNANTDIVNVSNNANKMFSINGTINDNSKVKGY  252
              +I    +  +   N K V TS Y    N N D+     N N+    N  +++ +  +
Sbjct  845   KRIKSSSVLNMRYKNDKYVDTSGYDSNININGDVYKYPTNKNQFGIYNDKLSEVNISQND  904

Query  253   LQEKDNKL--------IGNININDKGYNIQLDAIMEDALKTNLNSIKTTIINKNDHTFRG  304
                 DNK         +   N ++K  N+  +  + + ++ N +  K ++    +H
Sbjct  905   YIIYDNKYKNFSISFWVRIPNYDNKIVNVNNEYTIINCMRDNNSGWKVSL----NH----  956

Query  305   NTSIIDIRDNSGMTYIFKEITVDEFKEIIANDKSNNKNKQIVFELTNNKKCALDINSKDV  364
             N  I  ++DNSG+           F    AN  S+  NK I   +TN++         D
Sbjct  957   NEIIWTLQDNSGINQKLA------FNYGNANGISDYINKWIFVTITNDR-------LGDS  1003

Query  365   NLYISDEEYNSFDISDILNSPNI-IGEKVLSELKN--YQNYIGIK  406
              LYI     N  D   ILN  NI + + +L ++ N  Y  YIGI+
Sbjct  1004  KLYI---NGNLIDKKSILNLGNIHVSDNILFKIVNCSYTRYIGIR  1045
```
