# Supplementary figures and images for: Insights into the evolutionary origins of clostridial neurotoxins from analysis of the Clostridium botulinum strain A neurotoxin gene cluster
Source: BMC Evol Biol. 2008 Nov 14;8:316. doi: 10.1186/1471-2148-8-316 (PMC2605760; doi:10.1186/1471-2148-8-316)

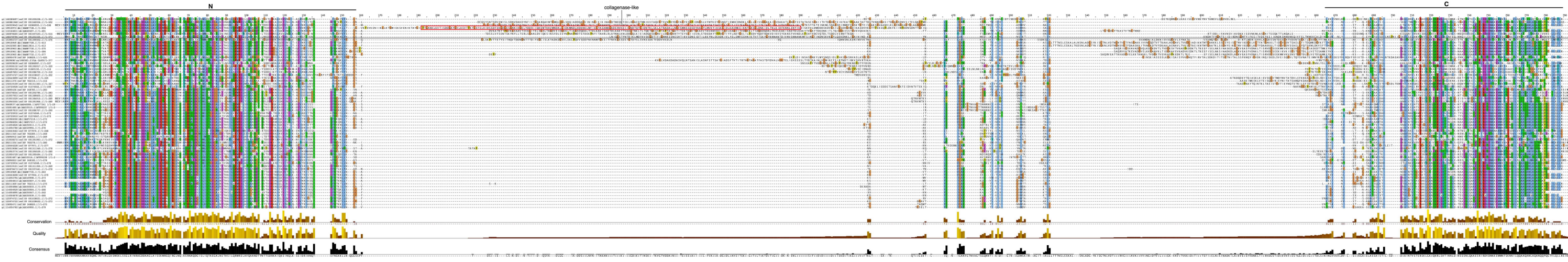

Supplement: Additional file 3 — Multiple alignment of clostridial flagellins. The N- and C-terminal domains are indicated, and the intermediate section represents the flagellin hypervariable region. The collagenase-like insert identified within the hypervariable region of FliA(H) is boxed in red. CBO0798 is underlined in black. Additional clostridial flagellins containing large hypervariable region inserts are grouped with CBO0798 and FliA(H) at the beginning of the alignment. [file 1471-2148-8-316-S3.pdf]
